# Lumen-Apposing Metal Stents for Palliation of Gastrointestinal Tumors

**DOI:** 10.3390/cancers17040600

**Published:** 2025-02-10

**Authors:** Pujitha Kudaravalli, Sahib Singh, Antonio Facciorusso

**Affiliations:** 1 Gastroenterology & Hepatology, Lahey Hospital & Medical Center, Burlington, MA 01803, USA; pkudaravalli17@gmail.com; 2 Department of Internal Medicine, Sinai Hospital of Baltimore, Baltimore, MD 21215, USA; sahibs559@gmail.com; 3 Gastroenterology Unit, Department of Experimental Medicine, Università del Salento, 73100 Lecce, Italy

Interventional endoscopy is gaining ground in the armamentarium of the management of cancer-related complications. EUS-guided therapies, with the placement of lumen-apposing metal stents (LAMSs), have revolutionized the management of patients with altered anatomy (such as gastric bypass, afferent limb syndrome), difficult biliary drainage, and gastric outlet obstruction (GOO). These approaches are also being increasingly used for a number of off-label indications, with a high degree of success. The ease of deployment and familiarity with the delivery system makes EUS-LAMSs an excellent option when conventional methods have failed.

LAMSs are self-expanding and fully covered devices that can form a stable anastomosis between adjacent organs and cavities. Among the different types of LAMS, including Axios (Boston Scientific, Marlborough, MA, USA), Spaxus and NAGI (Taewoong Medical Co., Goyang-si, Republic of Korea), Aixstent PPS (Leufen Medical, Berlin, Germany), and Hanarostent (M.I. Tech, Gyeonggi-do, Republic of Korea), Axios is the only one available for human use in the United States. Hot-Axios is shaped like a dumbbell or a saddle shape with terminal flanges, with an inner diameter (ID) of 6, 8, 10, 15, or 20 mm and a body length from 10 to 15 mm.

Malignant biliary obstruction can be caused by pancreatic ductal adenocarcinomas, cholangiocarcinomas, gallbladder adenocarcinomas, duodenal malignancies, lymphomas, or metastatic lymph nodes [1]. It leads to bile duct occlusion causing jaundice, debilitating symptoms, and pruritus. In such patients undergoing palliative care, endoscopic biliary decompression has become an essential method for symptom relief [2]. Establishing biliary drainage is a prerequisite to administering palliative chemotherapy, reduce the risk of cholangitis, and manage symptoms such as itching in those that are inoperable. Currently, endoscopic retrograde cholangiopancreatography (ERCP) is the gold standard for biliary drainage in these patients.

Percutaneous transhepatic cholangiography (PTC) has historically been the first-choice alternative when biliary drainage with ERCP has been unsuccessful. However, PTC of the gallbladder can have a morbidity rate of around 16% and adverse events in around 40% of cases in high-risk patients [3]. Significant pain is reported post PTC by patients, an external drain is less desirable by patients, and a recent meta-analysis has shown that EUS-guided drainage has fewer side effects [4]. Bile leak, peritonitis, catheter leak, bleeding, and fistula formation are other adverse events associated with PTC [Contribution 1]. The European Society of Gastrointestinal Endoscopy (ESGE) recently recommended the utilization of EUS-guided biliary drainage over PTC when ERCP has been unsuccessful [5]. Several studies demonstrated that EUS-BD with LAMS is a safe and effective procedure in patients who have had an unsuccessful ERCP [6,7]. Facciorusso et al. further assessed EUS-guided choledochoduodenostomy (EUS-CDS) and EUS-guided hepaticogastrostomy (EUS-HGS) and found no difference between the two both in terms of clinical success and adverse events [Contribution 2].

Vososu et al., in their review article, summarized the current literature comparing EUS-CDS with EUS-HGS. The review compared the different techniques used in performing the procedure, such as using electrocautery to reduce procedure times, limiting the use of different accessories, the size of stents, the use of X-ray versus ultrasound alone, the need for co-axial plastic stents, and wire-guided versus freehand methods. They further compared the efficacy and outcomes between different therapeutic options [Contribution 3].

EUS-GBD has been used successfully for patients with acute cholecystitis, who are poor candidates for surgery. In their systemic review, McDonagh et al. evaluated EUS-GBD in patients with malignant biliary obstructions. A total of 136 patients across seven retrospective studies were included. EUS-GBD was performed after ERCP failure in all of the studies, with EUS-BD being attempted before EUS-GBD in some cases. A 100% technical success rate was attained. The pooled rate of clinical success was 85%, which was described as a reduction in bilirubin of greater than 50% after two weeks in five studies and in the remaining studies as a significant improvement in liver enzymes. The adverse events were mild to moderate and were able to be managed conservatively. This study supported the use of EUS-GBD in cases where ERCP and EUS-BD have failed [Contribution 1]. EUS-GBD requires technical expertise and training, and with ongoing advancements in the field of echo-endoscopy, this could even challenge ERCP for being the standard of care based on emerging data and some studies that have already established similar efficacy. However, there is longer patency of the stents with the former approach [8,9].

EUS-guided gastroenterostomy is offered as a second-line option in most centers for the management of malignant gastric outlet obstruction. However, EUS-GE, as a first-line approach in certain patients, could avoid recurrent GOO after enteral stent placement, especially in patients with multiple strictures. It could also shorten the length of hospitalization, avoid delays in oncological therapy, and prevent multiple interventions under general anesthesia in frail patients [10]. In a study conducted by Enrique Perez-Cuadrado-Robles et al., clinical and technical success (defined as tolerating a solid diet and the creation of anastomosis, respectively), nutritional parameters and adverse events were compared between EUS-GE as first-line and EUS-GE as second-line therapy in those with prior enteral stenting. This is an observational single-center study using a prospectively collected database. Patients who underwent EUS-GE using the standardized WEST technique were included, of whom 13 had prior ES and 15 patients did not. Technical success was achieved in 25 cases (89.3%), with no differences between patients with or without a previous duodenal stent. Clinical success was achieved in 88% of the patients. Patients with a previous ES had quicker progression in the diet (GOOSS at 48 h, 2 vs. 1, *p* = 0.023). However, the two groups had comparable GOOSS at 1 week (*p* = 0.299), albumin gain (*p* = 0.366), and BMI gain (0.257). Two patients had severe and fatal AEs, with the overall AE rate being 7.1%. The severe AEs were not due to the duodenal stent. Over the median follow up of around 4 months, GOO recurrence occurred in four patients (18.2%) due to tumoral progression with secondary obstruction of LAMS and downstream peritoneal carcinomatosis. Thus, EUS-GE was shown to have high technical and clinical success in GOO regardless of previous ES, making it a potential first-line therapy to avoid unnecessary procedures and ES-related AEs [Contribution 4].

In a meta-analysis conducted by Emanuele Maria Rizzo et al., 11 studies with 337 patients with concurrent MBO and GOO were included. The mean technical and clinical success rates of EUS-BD were 96.4% (CI 95%, 92.18–98.99) and 84.96% (CI 95%, 67.99–96.26), respectively. Duodenal stenting was technically successful in all of the included studies. EUS-GEA showed technical success of 95.6%, with 100% clinical success, although it was only performed in one study. EUS-BD and EUS-GEA are options that need further exploration to determine whether they can be performed in the same session or during the same hospitalization for patients with combined MBO and GOO [Contribution 5].

Finally, Paduano et al. reviewed the role of EUS-BD in the palliation of malignant biliary obstructions [Contribution 6].

In conclusion, this Special Issue sheds light on EUS-guided procedures as alternatives in effectively managing malignant distal biliary obstruction and malignant GOO. However, these techniques, like many other recent advances in gastrointestinal endoscopy [11], need skilled endoscopists with extensive training in EUS and ERCP, as these procedures can be associated with serious adverse events. Further research is warranted to appropriately select patients for these procedures and standardize techniques.

## Data Availability

The data presented in the study are openly available in online databases.
